# Parental Beliefs and Actual Use of Corporal Punishment, School Violence and Bullying, and Depression in Early Adolescence

**DOI:** 10.3390/ijerph18126270

**Published:** 2021-06-10

**Authors:** Ji-Kang Chen, Zixin Pan, Li-Chih Wang

**Affiliations:** 1 Department of Social Work, Chinese University of Hong Kong, Hong Kong; ZixinPan@link.cuhk.edu.hk; 2 Department of Special Education, National Tsing Hua University, Hsinchu 300044, Taiwan; wanglc@mx.nthu.edu.tw

**Keywords:** corporal punishment, parenting, teachers’ aggression, victimization by teachers, bullying, depression, maltreatment, child abuse, school violence

## Abstract

Prior studies on adverse outcomes of parental corporal punishment on children have focused on examining one of two broad domains of parental corporal punishment: parental beliefs or actual use. Recently, researchers have argued that parental belief and actual use of corporal punishment should work jointly to contribute to children’s depression and involvement in school violence. Yet, studies supporting this proposition are lacking. This study examined the indirect link from parental attitudes towards corporal punishment to children’s depression and school violence involvement through actual use of corporal punishment. Four hundred and thirty-three elementary school students and their parents in Taiwan participated in this study. The results indicate that positive parental attitudes towards corporal punishment do not predict children’s depression and involvement in school violence. However, parental attitudes towards corporal punishment had significant indirect relationships with depression and involvement in school violence through the actual use of corporal punishment. These findings applied to both genders. This study supports the proposition that parental attitudes and the actual use of corporal punishment could work together to predict children’s depression and school violence. Future intervention programs for decreasing children’s depressive symptoms and involvement in school violence might need to tackle corporal punishment in the family.

## 1. Introduction

School violence and bullying as well as depression are of significant concern to the public worldwide, particularly among school-aged children [1,2,3,4]. Around 10–20% of children globally experience mental health problems and depression, one of the leading causes of illness and disability among children [5]. Nearly one in three children experienced at least one form of bullying and violence by school peers [6]. A recent report has also indicated that nearly 30% of children suffer from mental disorders in Taiwan, and around 3% have thought about taking their own lives [7]. In addition, school bullying and violence are widespread in Taiwan [8,9,10,11,12], and student victimization by peers, student perpetration against peers, student and maltreatment by teachers are three major forms negatively influencing Taiwanese school-aged students’ well-being [10,11,13,14,15]. 

Until now, numerous studies have been conducted to explore different factors associated with children’s depression, school violence, and bullying [9,11,12,14,15,16,17,18]. Parental corporal punishment, a common parenting practice to discipline children in many countries, particularly in East Asian cultures, such as Taiwan, has been theorized as a potential factor contributing to children’s negative psychological and behavioral outcomes [12,19,20,21,22]. However, relatively few empirical studies have been conducted to examine such direct links, and most of these studies have typically focused on examining one of two broad domains of parental corporal punishment: parental beliefs or actual use of corporal punishment [12,23,24,25,26]. 

Recently, researchers have argued that positive parental attitudes towards corporal punishment and actual use of corporal punishment are unlikely to act in isolation, and they should be considered jointly to identify risks for children’s psychological and behavioral problems, such as depression and school violence [27]. Several theories and interactive models provide potential frameworks to further understand how positive parental attitudes towards corporal punishment interact with their actual use to influence their children’s psychological and behavioral health. For example, the three-component model of parenting cognitions, parenting practice, and child adjustment [28] suggests that certain parental beliefs and values about child rearing, such as parental attitudes toward corporal punishment, guide their rearing practices (e.g., actual use of corporal punishment against children), which, in turn, influence their children’s psychological and behavioral outcomes (e.g., depression and school violence). However, few investigations have simultaneously studied the independent and joint contributions of parental beliefs and actual use of corporal punishment to children’s depression and involvement in school violence [27]; moreover, much fewer empirical studies have been conducted on the indirect influence of parental attitudes towards corporal punishment on children’s depression and involvement in school violence through actual parental use of corporal punishment.

Furthermore, previous studies on the associations of parental corporal punishment with children’s internalizing and externalizing problems relied primarily on parent surveys to examine research hypotheses [25]. It is problematic because previous studies have argued that parents’ reports of children’s internalizing and externalizing problems might not reflect children’s psychological and behavioral conditions accurately, decreasing the validity of these studies [12]. Recently, scholars have suggested adopting multiple reports (e.g., parents and children) to measure parenting and children’s outcome variables to prevent common-method variance and increase research validity [11,12,29,30,31]. However, empirical studies employing multiple perspectives to examine such links are still lacking. 

Using multiple pieces of information from parents and children, the current study aims to examine joint contributions of parental beliefs and actual use of corporal punishment to children’s depression and involvement in school violence and proposes a theoretical model to examine the indirect pathways from parental attitudes towards corporal punishment to children’s depression and involvement in school violence through actual parental use of corporal punishment.

### 1.1. Literature Review

A literature review indicates that theories and empirical studies on the associations between corporal punishment and negative effects on children have mainly explored or examined the direct associations of either parental attitudes/endorsement or actual use of corporal punishment on children’s external and internal problems in separate studies [12,23,24,25,26]. We briefly illustrate the relevant theories and studies in the following paragraphs. 

#### 1.1.1. Outcomes of Parental Actual Use of Corporal Punishment

Numerous theories and studies have suggested the direct link from the actual parental use of corporal punishment to children’s internal and external problems. For example, social control theory and social bonding perspectives have suggested that a weak bond with society enhances children’s motivations to engage in deviant behaviors, including school violence [32,33]. These theories suggest that the high quality of attachment with parents is one of the influential social bonds preventing children from further deviant behaviors [34,35]. Parental actual use of corporal punishment has been recognized as an aggressive act against children that may erode the affectionate attachment bond between parent and child [33,36]. Once parents use corporal punishment against their children, the strength of the bonds and relationships between parents and children may deteriorate, which in turn increases children’s risk of being involved in delinquent and violent behavior, such as school violence [20,33,37,38,39,40]. In addition, actual parental use of corporal punishment against children may lessen children’s sense of felt security in the family [41], enhancing children’s risk of suffering psychological distress, such as depression, anxiety, and fearfulness [42,43,44,45,46]. A substantial body of empirical studies from East and West has consistently shown a significant link from parental use of corporal punishment to toddlers’ and younger children’s general internal and external problems [47,48,49]. However, empirical studies examining the link from the actual parental use of corporal punishment to depression and school violence among school-aged children are still lacking. 

#### 1.1.2. Outcomes of Parental Beliefs about Corporal Punishment

Numerous theories have considered parental beliefs about corporal punishment as a risk factor in children’s behavioral and emotional problems. For example, emotional security theory suggests that parental aggression or negative parental attitudes, such as supportive beliefs about corporal punishment, may disrupt the children’s development of security and self-regulatory skills in childhood and lead to children’s emotional insecurity, increasing their risk of having emotional and behavioral problems, such as depression and involvement in school violence [50,51]. However, empirical studies on the link from positive parental attitudes towards corporal punishment to depression and school violence have been contradictory. Although most indicated significant associations [12,52,53], some did not [54]. The findings raised the question of whether certain psychosocial mechanisms influence the associations between parental beliefs in corporal punishment and adverse outcomes on children. 

### 1.2. Indirect Pathway through Parental Actual Use of Corporal Punishment

We argue that parental beliefs and actual use of corporal punishment are unlikely to act in isolation, and they should be considered jointly to identify risks for children’s psychological and behavioral problems, such as depression and school violence. Specifically, we hypothesize that the pathway from parental attitudes towards corporal punishment to children’s depression and involvement in school violence and bullying is indirect through parental use of corporal punishment. The three-component model of parenting cognitions, parenting practices, and child adjustment [28] provides the framework to support this proposition, which suggests that parental cognitions, such as beliefs and values about child-rearing practice, guide their child-rearing practice, which, in turn, determines their children’s behavioral and psychological outcomes [28]. Accordingly, parents who believe that corporal punishment is an effective parenting method are more likely to use corporal punishment against their children, enhancing children’s risk of psychological distress (e.g., depression) and involvement in negative behavioral outcomes, such as school violence and bullying. 

To the best of our knowledge, no empirical studies have employed the parenting cognitions-parenting practice-child adjustment model as a framework to examine how parental beliefs in corporal punishment indirectly influence children’s depression and involvement in school violence through the actual use of corporal punishment. Only one related study conducted by Fass et al. [25] found the indirect association of Arab mothers’ positive attitudes towards corporal punishment with their kindergarten children’s internalizing and externalizing behaviors through their actual use of punishment. However, that study relied only on mothers’ self-reports of their beliefs, actual use of corporal punishment, and their kindergarten children’s general internal and external problems, which may have led to inflated associations between variables due to shared source and method variance [11,12,55]. As a result, how parental beliefs in corporal punishment indirectly influence school-aged or older children’s depression and involvement in school violence through the actual use of corporal punishment is still unclear. 

### 1.3. Aims of the Current Study

In summary, based on the abovementioned review of the literature, the present study used multi-informant data from both parents and their children to examine how parents’ belief and actual use of corporal punishment work together to contribute to depression and involvement in school violence among early adolescence. Specifically, the present study examines a proposed theoretical model (guided by the three-component parenting cognitions-parenting practice-child adjustment model) of indirect effects of parents’ positive attitudes towards corporal punishment on children’s depression, violence against school peers, and victimization by school peers and teachers through parents’ actual use of corporal punishment. 

In addition, it has been argued that the interrelationship between parental beliefs about corporal punishment, actual use of corporal punishment, depression, and involvement in school violence may differ between boys and girls, because some of the previous studies showed gender differences in the impacts of corporal punishment on children’s psychological and behavioral outcomes [20,56,57]. In contrast, the parenting cognitions-parenting practice-child adjustment model assumes that the interrelations between parental belief/cognitions, parenting practice, and children’s psychological and behavioral outcomes hold for both genders [28]. Accordingly, the interrelationships between parental beliefs about corporal punishment, actual use of corporal punishment, depression, and involvement in school violence would be similar across genders. However, empirical evidence supporting such a proposition is lacking. Thus, the present study examined whether the proposed theoretical model would differ by a child’s gender. According to parenting cognitions-parenting practice-child adjustment model, we hypothesized no significant differences between boys and girls in the interrelationship between parental beliefs about corporal punishment, actual use of corporal punishment, depression, and involvement in school violence in this study. 

## 2. Materials and Methods

### 2.1. Participants and Procedures

The data were a part of a pilot study of a large-scale research project on school violence and bullying in Chinese societies [10,30,58,59]. The respondents were recruited from students in grades 4 to 6 of elementary schools and both parents in one Taiwanese county. A cluster random sampling strategy was employed in which 20 schools were first randomly selected from over 60 schools in this county. In each of the selected schools, one class from grades 4 to 6 was chosen randomly. All the students in the selected classes and both of their parents were invited to participate in the study. In Taiwan, parent–teacher conferences are commonly held in every school at the beginning of each semester. Both parents are invited to school to discuss their children’s learning and academic progress as well as the school’s teaching plan with school teachers. Concerning the parental sample, questionnaires for parents were distributed by professionally trained survey monitors to both parents of selected students in a waiting room before parent–teacher meetings held at the beginning of the spring semester of 2016. The parent survey comprised items concerning basic demographics and other personal perspectives on parenting. It took about 5–10 min to complete the survey. 

Regarding the student sample, students were given a questionnaire in classrooms under the guidance of professionally trained research assistants at the end of the spring semester in 2016 (around 4–5 months after parent–teacher meetings or conferences). The student questionnaire included items assessing students’ personal and school experiences. It took about 35 min to complete the survey.

Written consent was obtained from school principals and teachers, students, and parents before administering the surveys. The ethics committee of the first author’s university reviewed and approved the questionnaires, procedures, and informed consent forms. 

A total of 491 students and their parents participated in the study. We excluded 58 students from single-parent families or two-parent families if only one parent returned the completed surveys. Next, we paired students with both of their parents. As a result, our final sample consisted of 433 parent–child triads. Of this sample, 214 (49.4%) students were boys, and 219 (50.6%) were girls.

### 2.2. Measurement

#### 2.2.1. Parental Beliefs about Corporal Punishment

One item assessed each parent’s beliefs about corporal punishment of their children on a five-point response scale (1 = strongly disagree to 5 = strongly agree). This item was “I believe that corporal punishment is the effective means of child discipline.” We constructed a latent variable of parental beliefs about corporal punishment using two items: father’s beliefs (factor loading = 0.65) and mother’s beliefs (factor loading = 0.73). 

#### 2.2.2. Actual Use of Physical Punishment

Eighteen items were administered to students to evaluate their parents’ use of corporal punishment to discipline them during the semester, with nine items assessing mothers and nine items assessing fathers. These items included common forms of physical punishment against children in Taiwan, such as spanking, slapping, hitting with rods/belts/other objects, kicking, beating, pinching, pushing, seizing, and grabbing. These items were derived from a scale used in previous large-scale surveys in Taiwan to assess different forms of punishment against children [60,61]. The responses were measured on a five-point Likert scale (1 = never to 5 = all the time). Due to the skewed distribution, each item of this scale was dichotomized as 0 (never) and 1 (at least one time). All dichotomized items were summed to indicate the actual use of corporal punishment. Greater scores indicated a higher level of actual use of corporal punishment. In this study, a latent variable of actual parental use of physical punishment was constructed by two factors/subscales: father’s actual use (factor loading = 0.87) and mother’s actual use (factor loading = 0.93).

#### 2.2.3. Student Victimization by Students

Student victimization by students was measured using a five-item scale assessing children’s exposure to peer violence in school during the semester. These five items, selected from a traditional Chinese version of the California School Climate and Safety Survey (CSCSS), asked student participants to rate on a five-point Likert scale ranging from 1 (never) to 5 (over 7 times) how frequently they were cursed, verbally insulted, hit/kicked/beaten, socially excluded, and threatened/blackmailed by schoolmates [10,11,13,29,30,62]. Because of a skewed distribution, each item was dichotomized as 0 (never) and 1 (at least one time). All dichotomized items were summed to obtain the scores of student victimization by peers. Greater scores indicated a higher level of student exposure to peer violence in school.

#### 2.2.4. Student Perpetration against Students

Student perpetration was assessed with a five-item scale, asking students whether they bullied their peers in school during the semester. The five questions, selected from a traditional Chinese version of CSCSS, asked students to rate on a five-point Likert scale ranging from 1 (never) to 5 (over 7 times) how frequently they cursed, insulted, hit/kicked/beat, excluded, and threatened/blackmailed other schoolmates [10,15]. Because of a skewed distribution, each item of this scale was dichotomized as 0 (never) and 1 (at least one time). All dichotomized items were summed to obtain the scores of student perpetration against students. Greater scores indicated a higher level of perpetration against school peers.

#### 2.2.5. Maltreatment by School Teachers

Three items selected from a traditional Chinese version of CSCSS measured the frequencies of children’s maltreatment by teachers during the semester on a five-point Likert scale ranging from 1 (never) to 5 (over 7 times) [11,12,13,14]. The three items asked children how frequently they were hit, kicked, beaten, slapped, cursed, humiliated, mocked, insulted, and publicly satirized by teachers in school. Because of a skewed distribution, each item of this scale was dichotomized as 0 (never) and 1 (at least one time). All dichotomized items were summed to determine the level of maltreatment by teachers. Greater scores indicated a higher level of maltreatment by teachers.

#### 2.2.6. Depression

A latent variable of depression was assessed by three items selected from a subscale of depression in the Brief Symptoms Rating Scale [11,12,58,63,64] measured on a 5-point Likert scale ranging from 1 (not at all) to 5 (very severe). These three items evaluated depressive mood (factor loading = 0.75), worthlessness (factor loading = 0.87) and hopeless (factor loading = 0.79). Cronbach’s alpha for these three items was 0.88. 

### 2.3. Plan of Analysis

Descriptive analyses of the variables in this study were conducted first, followed by a latent variables structural equations modeling (SEM) with maximum likelihood estimation tested using the AMOS 25.0 [65]. A bootstrapping approach (*n* = 2000 bootstrap samples) was used to evaluate the indirect effects of parental beliefs about corporal punishment on dependent variables through the actual use of corporal punishment [66]. Cross-group SEM was applied to examine gender differences in the theoretical model. The model fit was evaluated using the chi-square (χ^2^) difference test, which was expected to be non-significant; SEM incremental fit indices, including Normed Fit Index (NFI), Incremental Fit Index (IFI), and Confirmatory Fit Index (CFI), with values greater than 0.95 indicating a good model fit [67,68,69]; and the root mean square error of approximation (RMSEA), with values of less than 0.06 [70].

## 3. Results

### 3.1. Descriptive Statistics

Table 1 shows the means and standard deviations for the study variables broken down by gender. The correlations between variables are shown in Table 2. The results show a positive correlation between beliefs about corporal punishment and actual use of corporal punishment (*r* = 0.16, *p* < 0.01). Parental use of corporal punishment correlated positively with student victimization by students (*r* = 0.27, *p* < 0.01), student perpetration against students (*r* = 0.24, *p* < 0.01), maltreatment by teachers (*r* = 0.14, *p* < 0.01), and depression (*r* = 0.34, *p* < 0.01). The bivariate correlations between victimization by students, perpetration against students, maltreatment by the teacher, and depression were all positively related, with Pearson’s r values ranging from 0.12 to 0.54. Parental beliefs about corporal punishment were not correlated with victimization by students, perpetration against students, maltreatment by the teacher, and depression in this study (Pearson’s r values ranged from −0.03 to 0.01).

### 3.2. Overall Model

**Figure 1:**
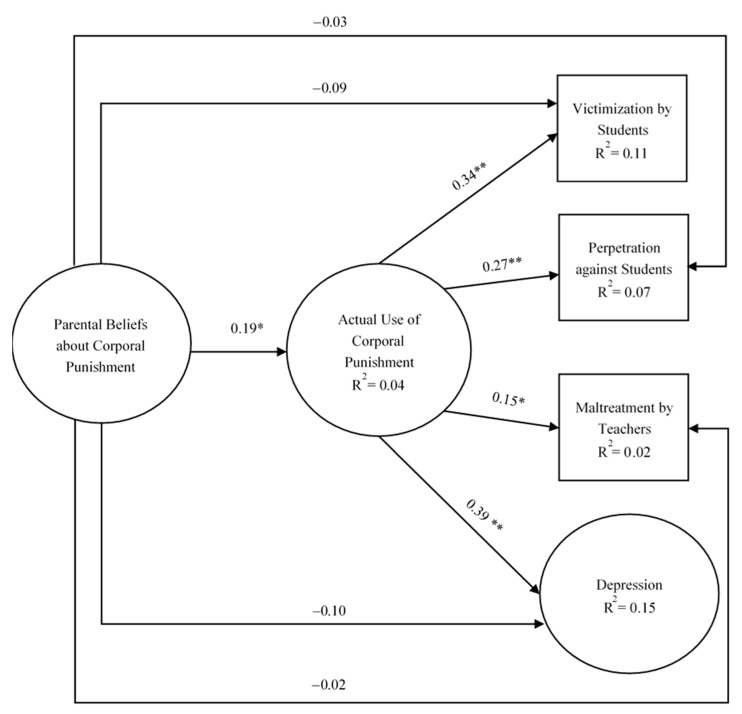
Overall model. Note. ** *p* < 0.001; * *p* < 0.01.

Based on the overall sample, the model analysis results show a good fit to the data, *χ*^2^ (23) = 27.00, *p* > *0*.05, NFI = 0.980, IFI = 0.997, CFI = 0.997, and RMSEA = 0.020. Figure 1 demonstrates the paths in this model. 

It shows that parental beliefs about corporal punishment had no significant direct association with victimization by students, perpetration against students, maltreatment by teachers, and depression (β = −0.09, β = −0.03, β = −0.02, and β = −0.10, respectively). However, the indirect association of parental beliefs about corporal punishment with victimization by students, perpetration against students, maltreatment by teachers, and depression through actual use of corporal punishment was significant. Overall, actual use of corporal punishment was a significant predictor of victimization by students, perpetration against students, maltreatment by teachers, and depression in this model (β = 0.34, β = 0.27, β = 0.15, and β = 0.39, respectively).

We generated 2000 bootstrapping samples from the original dataset by random sampling to evaluate the indirect effect. The results reveal that the indirect effects of parental beliefs about corporal punishment on victimization by students, perpetration against students, maltreatment by teachers, and depression through actual use of corporal punishment were, respectively, 0.071 (SE = 0.027, CI = [0.029, 0.139], *p* < 0.01), 0.057 (SE = 0.022, CI = [0.023, 0.116], *p* < 0.01), 0.031 (SE = 0.014, CI = [0.011, 0.072], *p* < 0.01), and 0.082 (SE = 0.030, CI = [0.036, 0.158], *p* < 0.01). The 95% confidence interval did not contain zero, signifying that parental beliefs about corporal punishment had a significant indirect association on all dependent variables via actual use of corporal punishment. 

All variables in this model contributed 11% of the explained variance to the victimization by students (R^2^ = 0.11), 7% to perpetration against students (R^2^ = 0.07), 2% to perpetration against students (R^2^ = 0.02), and 15% to depression (R^2^ = 0.15), which suggested that the overall model explained depression better than other dependent variables. 

### 3.3. Gender Comparison

**Figure 2:**
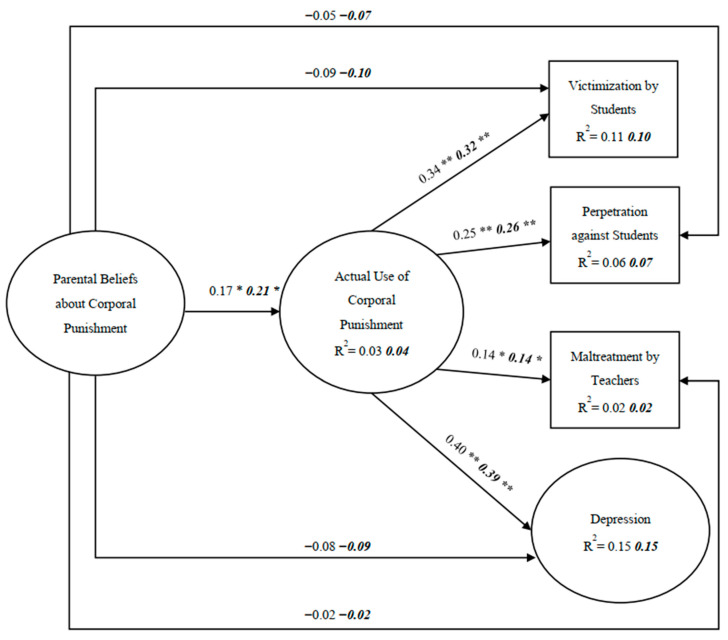
Sex comparison. *Note*. The coefficients in regular print and those in bold italics represent the results for boy and girl samples, respectively. ** *p* < 0.001; * *p* < 0.01.

In this multi-group SEM analysis, factor loadings and structural paths in this model were first constrained to be equal. The results reveal that the model fit indices were acceptable: *χ*^2^ (59) = 72.316, *p* > *0*.05, NFI = 0.947, IFI = 0.990, CFI = 0.989, and RMSEA = 0.023. Next, equality constraints on the structural paths were released one at a time but did not produce a significantly enhanced fit. The final unconstrained model fit the data well, with *χ*^2^ (50) =56.753, *p* > *0*.05, NFI = 0.958, IFI = 0.995, CFI = 0.995, and RMSEA = 0.018. Chi-square differences between constrained and unconstrained models showed no significant differences (Δ*χ*^2^ (9) = 15.563, *p* > 0.05), which indicated that no gender differences were found in the model. Figure 2 presents the results of this analysis, which indicates that the regression coefficients between genders for each path and the explained variance accounting for each dependent variable for both genders were similar.

## 4. Discussion

Using multiple pieces of information from parents and children, this study examined joint contributions of parental beliefs and actual use of corporal punishment to children’s depression and involvement in school violence. Specifically, this study examined the indirect pathways from parental beliefs about corporal punishment to children’s psychological distress and involvement in school violence through actual parental use of corporal punishment. We also examined whether the abovementioned indirect pathways differ by gender.

### 4.1. Overall Model

The results show significant direct links from the actual parental use of corporal punishment to student victimization by schoolmates, perpetration against school peers, maltreatment by teachers, and depression. The findings support the social control theory and social bonding perspectives that the deteriorated or broken attachment bonds between parents and children resulting from the actual parental use of corporal punishment reinforce children’s motivations to engage in school violence and increase children’s risk of being exposed to violence in school [12,30,33,37,38,39,40]. In addition, the findings support the proposition that actual parental use of corporal punishment against children may lessen children’s sense of felt security in the family [41], enhancing children’s risk of suffering psychological distress, such as depression [42,43,44,45,46]. The findings provide further evidence that the actual use of corporal punishment plays a role in children’s involvement in school violence and depression. Although our results show a significant association between parental beliefs and actual use of corporal punishment, the regression coefficient is not large. The findings are in line with previous studies showing a weak or moderate relation between attitudes and behaviors in parenting [27,28,71]. The findings may imply that parents’ beliefs in parenting do not map onto practices all the time, although the two variables are significantly related [72].

Our results show that direct associations of parental beliefs about corporal punishment with student victimization by schoolmates, student perpetration against students, maltreatment by teachers, and depression are not significant, which is consistent with previous research reports showing non-significant relationships between parental endorsement of corporal punishment and children’s externalizing and internalizing problems [54]. However, it does not mean that parental beliefs about corporal punishment are not important in affecting children’s depression and involvement in school violence, because the results of this study show that positive parental beliefs about corporal punishment have an indirect link with student victimization by schoolmates, student violence against school peers, maltreatment by teachers, and depression through parental use of corporal punishment. The findings suggest that parents who believe that corporal punishment is an effective way of parenting are more likely to use corporal punishment against their children, which increases their children’s risk of victimization by schoolmates, bullying other school peers, maltreatment by teachers, and depression. These findings are consistent with other studies showing that positive parental attitudes towards corporal punishment and actual use of corporal punishment are unlikely to act in isolation, and they should be considered jointly to identify risks of developing psychological and behavioral problems, such as depression and school violence [27]. Furthermore, the findings are consistent with the parenting cognitions-parenting practice-child adjustment model, which proposes that parents’ beliefs and values about child rearing (i.e., positive attitudes towards corporal punishment) guide their rearing practice (i.e., actual use of corporal punishment), influencing children’s psychological and behavioral outcomes or adjustment problems, such as depression and school violence.

Parental beliefs and actual use of corporal punishment in this study explained 15% of the variance in depression, 11% in victimization by students, 7% in perpetration against students, and 2% in maltreatment by teachers. The overall model explained children’s depression better than victimization by students, perpetration against school peers, and maltreatment by teachers. These findings may imply that parental corporal punishment has stronger negative outcomes on children’s depression compared to student involvement in school violence.

### 4.2. Gender Differences

The results do not reveal a significant difference between boys and girls in the proposed model. Thus, the model applies to both boys and girls. Additionally, the results show that the interrelationships of parental beliefs with the actual use of corporal punishment, student victimization by schoolmates, perpetration against peers, maltreatment by teachers, and depression between boys and girls were similar. The findings are consistent with the parenting cognitions-parenting practice-child adjustment model in which interrelations between parental belief/cognitions, parenting practice, and children’s psychological and behavioral outcomes were robust to boys and girls [28]. Furthermore, the previous studies have shown no buffering effect of gender on the association of corporal punishment with children’s externalizing and internalizing behaviors [25]. These findings may imply that regardless of the differences between boys and girls in the prevalence of parental corporal punishment, school violence, and depression, the interrelationship between these variables and the indirect effects of positive parental attitudes towards corporal punishment on school violence and depression do not differ by gender. 

### 4.3. Limitations

Several limitations of this study need to be considered when interpreting the results. First, the study did not control for time effects when examining the associations between the variables. Thus, the results cannot be used to build causal relationships. Future studies may use panel data and cross-lagged analysis to provide further evidence of causal associations of parental beliefs with the actual use of corporal punishment, school violence, and children’s depression. Second, 433 pairs of both parents and their children participated in this study, and this sample size is relatively small. Larger scale survey studies are encouraged in the future to replicate our findings. Third, this study used a random sample of primary school students in early adolescence in Taiwan. The results may not be generalizable to other age groups or cultural contexts. Future studies could replicate this model in other age groups and countries to confirm our findings. Fourth, we asked students to evaluate their parents’ use of corporal punishment in this study. Future studies may consider collecting information on this variable from other sources, such as parents, to increase research validity. Finally, only one item asked fathers and mothers about their beliefs regarding corporal punishment in this study. Future research may construct more items to assess parental attitudes towards corporal punishment more accurately and increase the research validity.

## 5. Conclusions

In contrast to previous studies on negative outcomes of corporal punishment typically focusing on examining how either parental beliefs or actual use of corporal punishment affect children’s internal and external problems, this study provides evidence to support a joint effect of parental attitudes and actual use of corporal punishment on children’s psychological and behavioral outcomes. Specifically, the findings of this study indicate that the effects of positive parental attitudes towards corporal punishment on children’s depression and involvement in school violence are dependent on whether corporal punishment is inflicted on children by their parents. Accordingly, intervention programs for decreasing Taiwanese children’s depressive symptoms and involvement in school violence might consider tackling corporal punishment in the family. 

Although Taiwan has relatively comprehensive laws and advanced intervention programs for dealing with family child abuse, most parents still believe that they have the right to psychologically and corporally punish their children, and “appropriate” psychological and corporal punishment by parents is commonly acceptable and lawful [12,73]. Our findings highlight the negative effects of parental corporal punishment on children’s depression and involvement in school violence. Thus, we urgently call for practitioners and organizations working for children’s rights in Taiwan to advocate governments and legislators for law reforms prohibiting psychological and corporal punishment in the family to decrease the incidents of parental corporal punishment and further prevent children from developing internal and external behavior problems, such as depression and school violence and bullying. Furthermore, family practitioners in Taiwan should promote parenting education programs to change parents’ beliefs, hinder their use of corporal punishment, and help them adopt positive ways to educate and discipline their children. Intervention programs could further focus on enhancing parents’ knowledge and skills about child rearing. For example, previous studies have suggested that the group-based parent education programs, such as the *Adults and Children Together Against Violence* educational program and *Positive Discipline in Everyday Parenting* program, have been effective in teaching parents about nonviolent discipline, child development, and non-punitive problem-solving skills, in shifting parents’ attitudes toward corporal punishment, and in reducing their actual use of corporal punishment [74,75,76,77]. Meanwhile, children’s rights organizations, communities, and governments in Taiwan may seriously consider launching public education or national campaigns about the risk of corporal punishment and the benefits of alternative nonviolent discipline strategies via various media platforms (e.g., TV or the Internet) or via written content (books and posters) because previous studies have shown that these strategies can efficiently affect parental changes in attitude and use of corporal punishment [74,78].

## Figures and Tables

**Table 1:** Means and standard deviations of each scale by sex groups (standard deviations in parenthesis).

	Overall	Sex Groups	
		Male	Female
Parental beliefs about corporal punishment ^a^	6.17	6.32	6.03
	(1.81)	(1.74)	(1.87)
Parental actual use of corporal punishment ^b^	3.65	4.37	3.03
	(3.99)	(4.22)	(3.68)
Victimization by students ^b^	1.29	1.34	1.25
	(1.38)	(1.40)	(1.36)
Perpetration against students ^b^	0.79	0.90	0.68
	(1.08)	(1.17)	(0.98)
Maltreatment by teachers ^b^	0.21	0.24	0.18
	(0.58)	(0.60)	(0.56)
Depression ^c^	5.60	5.60	5.59
	(2.70)	(2.78)	(2.64)

*Note*. ^a^ On a scale: from 1 = strongly disagree to 5 = strongly agree. ^b^ On a scale: 0 = never and 1 = at least one time. ^c^ On a scale: 1 = not at all to 5 = very severe.

**Table 2:** Intercorrelations between variables in the model.

	1	2	3	4	5	6
1. Beliefs about corporal punishment	--	0.16 **	−0.03	0.01	0.01	−0.02
2. Actual use of corporal punishment		--	0.27 **	0.24 **	0.14 **	0.34 **
3. Victimization by students			--	0.54 **	0.35 **	0.29 **
4. Perpetration against students				--	0.34 **	0.24 **
5. Maltreatment by teachers					--	0.12 *
6. Depression						--

*Note*. ** *p* < 0.01; * *p* < 0.05.

## Data Availability

The data presented in this study are available on request from the corresponding author and with permission of the Survey and Behavioural Research Committee, Chinese University of Hong Kong.
